# Dynamic behavior of DNA topoisomerase IIβ in response to DNA double-strand breaks

**DOI:** 10.1038/s41598-018-28690-6

**Published:** 2018-07-09

**Authors:** Keiko Morotomi-Yano, Shinta Saito, Noritaka Adachi, Ken-ichi Yano

**Affiliations:** 1 Department of Bioelectrics, Institute of Pulsed Power Science, Kumamoto University, Kumamoto, 860-8555 Japan; 2 Department of Life and Environmental System Science, Graduate School of Nanobioscience, Yokohama City University, Yokohama, 236-0027 Japan; 3 Advanced Medical Research Center, Yokohama City University, Yokohama, 236-0004 Japan

## Abstract

DNA topoisomerase II (Topo II) is crucial for resolving topological problems of DNA and plays important roles in various cellular processes, such as replication, transcription, and chromosome segregation. Although DNA topology problems may also occur during DNA repair, the possible involvement of Topo II in this process remains to be fully investigated. Here, we show the dynamic behavior of human Topo IIβ in response to DNA double-strand breaks (DSBs), which is the most harmful form of DNA damage. Live cell imaging coupled with site-directed DSB induction by laser microirradiation demonstrated rapid recruitment of EGFP-tagged Topo IIβ to the DSB site. Detergent extraction followed by immunofluorescence showed the tight association of endogenous Topo IIβ with DSB sites. Photobleaching analysis revealed that Topo IIβ is highly mobile in the nucleus. The Topo II catalytic inhibitors ICRF-187 and ICRF-193 reduced the Topo IIβ mobility and thereby prevented Topo IIβ recruitment to DSBs. Furthermore, Topo IIβ knockout cells exhibited increased sensitivity to bleomycin and decreased DSB repair mediated by homologous recombination (HR), implicating the role of Topo IIβ in HR-mediated DSB repair. Taken together, these results highlight a novel aspect of Topo IIβ functions in the cellular response to DSBs.

## Introduction

DNA topoisomerase II (Topo II) is an ATP-dependent enzyme that resolves DNA topological problems, such as supercoiling and catenation^1^. In eukaryotes, Topo II plays important roles in various cellular processes, including DNA replication, transcription, and chromosome condensation and segregation, all of which can give rise to topological constraints of chromosomal DNA^2^. Topo II is composed of three domains, that is, an ATPase domain in the N-terminus, a central catalytic domain, a C-terminal domain^3^. Topo II functions as a homodimer that forms a clamp-like structure^4^. In the first step of its catalytic reaction cycle, Topo II binds to two DNA duplexes, then transports one DNA duplex through another by generating a transient DNA double-strand break (DSB), and then finally religates the DNA ends^5^. A number of drugs targeting specific steps in the Topo II catalytic cycle have been developed, most of which fall into two classes, namely Topo II poisons and Topo II catalytic inhibitors. Topo II poisons, such as etoposide, halt the Topo II catalytic reaction cycle during formation of a covalent Topo II-DNA complex, which is readily converted into a DSB in living cells^5^. While Topo II poisons produce DSBs, Topo II catalytic inhibitors block the catalytic cycle without a marked increase in DSB production^5^. For example, ICRF-187 and ICRF-193 halt the catalytic cycle by trapping Topo II in the closed clamp structure, in which a DNA duplex is captured^6^.

In mammals, there are two Topo II isozymes, Topo IIα and Topo IIβ, which share striking sequence homology with one another in their N-terminal ATPase and central catalytic domains but differ in their C-terminal domains^7^. Although these two isozymes have similar enzymatic properties *in vitro*^8^, they exhibit characteristic expression patterns and play distinct roles in cellular processes. Topo IIα is highly expressed in proliferating cells with peak expression at late S and G2/M phases of the cell cycle^9^. Topo IIα primarily functions in the resolution of DNA topology problems that arise during DNA replication and mitosis^10^. On the other hand, Topo IIβ is expressed in both dividing and non-dividing cells^9,11^, and its function is largely dispensable for cell cycle progression^12,13^. Although the role of Topo IIβ in cellular processes is ambiguous as compared to Topo IIα, previous studies have implicated Topo IIβ in the transcription of subsets of genes, such as developmentally-controlled genes^14,15^ and hormone-regulated genes^16–18^.

Interestingly, in the transcriptional control of a subset of hormone-responsive genes, Topo IIβ interacts with proteins that function in the repair of DSBs^16–18^. DSBs are the most lethal form of DNA damage, and its induction rapidly causes cell cycle arrest^19^. Ataxia telangiectasia mutated (ATM) protein, a member of the phosphoinositol 3-kinase-related kinase (PI3KK) family, plays a central role in signal transduction during this process^20^. ATM is activated by DSB induction and in turn undergoes autophosphorylation at DSB sites^21^. Activated ATM influences DSB repair by phosphorylating multiple nuclear proteins. In mammalian cells, DSBs are primarily repaired by one of two major repair mechanisms. Homologous recombination (HR) functions during the late S to G2 phases of the cell cycle, since HR utilizes an intact sister chromatid as a repair template^22^. Non-homologous end-joining (NHEJ) rejoins two DSB ends and does not require a repair template. NHEJ is thus active throughout the cell cycle, and DNA-dependent protein kinase (DNA-PK) plays a critical role in this mechanism^23^. DNA-PK is composed of a Ku70-Ku80 heterodimer and a catalytic subunit (DNA-PKcs). Similar to ATM, DNA-PKcs belongs to the PI3KK family, and its activation manifests as autophosphorylation. In addition to HR and NHEJ, chromatin remodeling is also important in DSB repair. For example, several histone deacetylase (HDAC) members are recruited to DSB sites and are involved in chromatin remodeling during DSB repair^24,25^. Poly(ADP-ribose) polymerase-1 (PARP-1) is rapidly recruited to DSB sites and modifies histones and chromatin proteins with poly(ADP-ribose) to facilitate DSB repair^26^. Previous studies have shown that Topo IIβ forms a large complex with DNA-PKcs, Ku, and PARP-1 on the promoters of estrogen-, insulin-, and glucocorticoid-responsive genes^16–18^.

Topo II has attracted considerable attention as a therapeutic target because of its capability to induce DSBs in conjunction with Topo II poisons. However, it remains unclear how Topo II itself responds to DSB induction. Furthermore, although previous studies have demonstrated that Topo II participates in various cellular processes associated with DNA topology problems, such as replication and mitosis, limited information is available regarding the involvement of Topo II in DSB repair, which may also require the control of DNA topology. Given the observations on the association of Topo IIβ with DSB repair factors in transcriptional control^16–18^, we aimed to explore the response of Topo IIβ to DSB induction. Here, we show the spatiotemporal dynamics of human Topo IIβ in response to DSB induction. We observed the rapid recruitment of Topo IIβ to laser-induced DSB sites. Topo IIβ recruitment to DSB sites was partially abrogated by inhibitors for PARP-1 and HDAC, but, contrary to our assumption, it did not require DNA-PKcs or ATM. The Topo II catalytic inhibitors ICRF-187 and ICRF-193 prevented Topo IIβ recruitment to DSBs by reducing Topo IIβ mobility. Furthermore, Topo IIβ knockout cells exhibited increased sensitivity to bleomycin and reduced HR activity. These observations provide novel insight into the participation of Topo IIβ in the cellular response to DSB induction.

## Results

### Recruitment of endogenous Topo IIβ to laser-damaged sites

**Figure 1:**
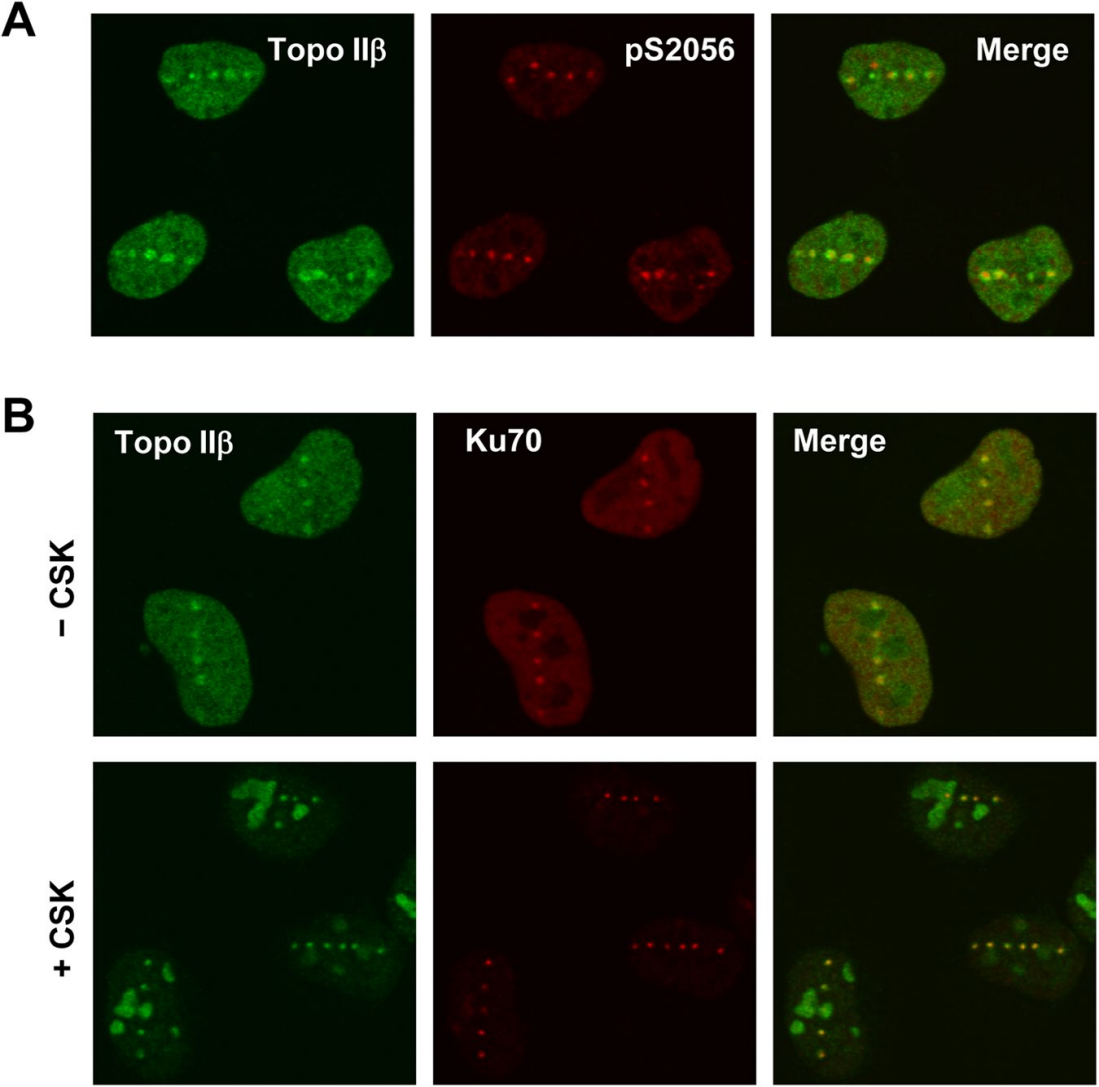
Topo IIβ is recruited to laser-damaged sites. (**A**) Recruitment of Topo IIβ to damaged sites. DNA damage was induced by pulsed UVA laser microirradiation of the nucleus of HeLa cells. After 10 min incubation at 37 °C, the cells were fixed and subjected to coimmunostaining for Topo IIβ and phosphorylated serine 2056 (pS2056) of DNA-PKcs. (**B**) Tight association of Topo IIβ with damaged chromatin. DNA damage was induced by pulsed UVA laser microirradiation. (Upper) The cells were fixed and coimmunostained for Ku70 and Topo IIβ. (Lower) The cells were treated with CSK buffer containing 0.5% Triton X-100 and subsequently subjected to fixation followed by immunostaining for Topo IIβ and Ku70.

In this study, we employed microirradiation of the nucleus using a pulsed UVA laser (349 nm) for induction of localized DNA damage. Compared to laser beams used for scanning confocal microscopy, a pulsed UVA laser can achieve high energy and thereby produce both DSBs and single strand breaks (SSBs)^27–29^. Microirradiation of the nucleus with a single shot of the pulsed UVA laser results in the production of DNA damage in a small punctate area. To investigate the effects of DNA damage on the nuclear distribution of endogenous Topo IIβ, we performed microirradiation of the nucleus of HeLa cells using a pulsed UVA laser. Laser-irradiated cells were subjected to fixation followed by immunostaining for endogenous Topo IIβ and phosphorylated DNA-PKcs, which served as a marker for induced DNA damage^30^. As shown in Fig. 1A, we observed punctate foci of endogenous Topo IIβ in the irradiated nucleus. Topo IIβ foci were colocalized with those of phosphorylated DNA-PKcs, indicating that endogenous Topo IIβ was recruited to damaged sites. Next, we performed immunostaining of HeLa cells that were treated with neocarzinostatin (NCS), a radiomimetic agent. As shown in Supplementary Fig. 1, we could not detect clear foci of Topo IIβ in NCS-treated cells, suggesting that Topo IIβ binds to damaged sites at relatively low abundance.

Nuclear proteins involved in DNA damage responses often display higher affinity for damaged chromatin than undamaged one, which manifests as the formation of detergent-resistant foci at damaged sites^31^. To further investigate the recruitment of Topo IIβ to damaged sites, laser-irradiated cells were treated with detergent-containing buffer to remove nuclear proteins in undamaged area. Following fixation, the detergent-extracted cells were subjected to immunofluorescence analysis of Topo IIβ. For comparison, the same samples were coimmunostained for Ku70, which is known to tightly associate with DSBs^23^. As shown in Fig. 1B, endogenous Topo IIβ and Ku70 were retained as foci in detergent-extracted cells and colocalized with each other. Both proteins in the undamaged nucleoplasm were washed out by detergent treatment, supporting the tight association of endogenous Topo IIβ with damaged sites. In several detergent-treated cells, Topo IIβ resided in the nucleoli, presumably because alterations in cellular energy status arose during detergent treatment and consequently caused stable accumulation of Topo IIβ therein as previously reported^32^.

### Live imaging of EGFP-Topo IIβ recruitment to DSBs

**Figure 2:**
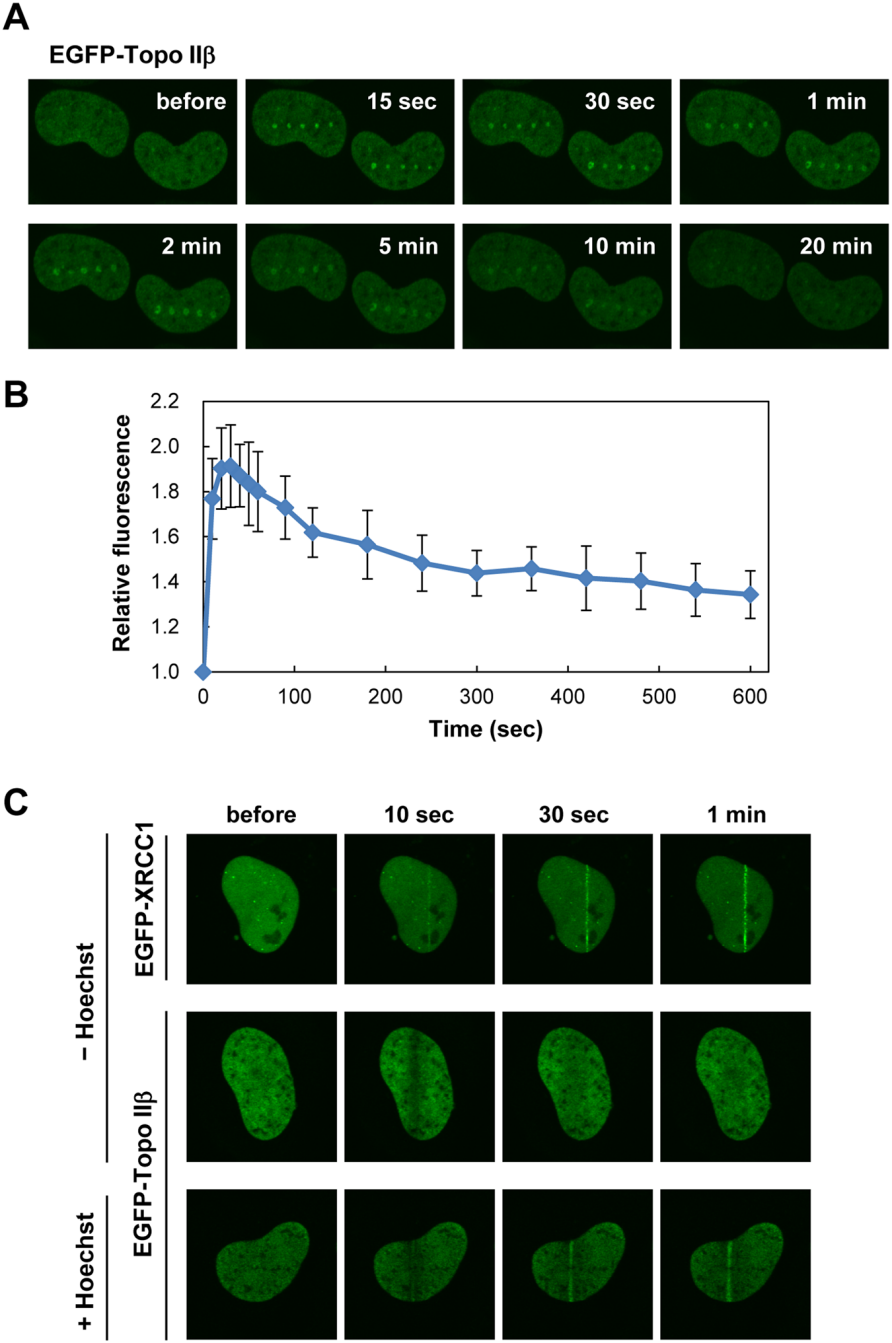
Live imaging of Topo IIβ recruitment to damaged sites. (**A**) Recruitment of EGFP-Topo IIβ to damaged sites. EGFP-Topo IIβ was transiently expressed in HeLa cells. DNA damage was induced with shots of a pulsed UVA laser, and recruitment of EGFP-Topo IIβ to the damaged sites was monitored by time-lapse microscopy. Representative images at the indicated time points are shown. (**B**) Time-course of EGFP-Topo IIβ recruitment to damaged sites. DNA damage was induced in the nucleus with a single shot of a pulsed UVA laser. After time-lapse imaging, EGFP signals in the damaged site were quantified, and average values with SD were calculated from 10 cells. (**C**) Recruitment of EGFP-Topo IIβ to DSB sites. Either EGFP-XRCC1 or EGFP-Topo IIβ was transiently expressed in HeLa cells. The cells were line-scanned with a 405 nm laser in the presence or absence of Hoechst 33342. Representative images at the indicated time points are shown.

We next examined the recruitment of Topo IIβ to damaged sites in living cells. Human Topo IIβ fused to EGFP was transiently expressed in HeLa cells, and DNA damage was induced by microirradiation with a pulsed UVA laser. As shown in Fig. 2A, EGFP-Topo IIβ was recruited to the damaged sites immediately after laser microirradiation. Recruitment of EGFP-Topo IIβ was transient, and fluorescent signals for EGFP-Topo IIβ foci gradually became faint and then marginally detectable after 20 min. Quantification analysis highlighted the fast kinetics of Topo IIβ recruitment to damaged sites with a peak observed within a minute (Fig. 2B).

Although the microirradiation with a pulsed UVA laser is known to induce both DSBs and SSBs^27–29^, we predicted that Topo IIβ was recruited to DSB sites, owing to the observed colocalization of Topo IIβ with phosphorylated DNA-PKcs (Fig. 1A) and Ku70 (Fig. 1B) at laser-induced foci. To test this notion, DSBs and SSBs were produced by 405 nm laser line-scanning in the presence and absence of Hoechst 33342, respectively. Line-scanning of the nucleus with a 405 nm laser produces SSBs, but not DSBs, due to its insufficient energy^27,28^. Hoechst 33342 serves as a photosensitizer that enables the production of DSBs by 405 nm laser line scanning^27,28^. First, we confirmed the production of SSBs after 405 nm laser scanning without Hoechst 33342 by examining the localization of EGFP-XRCC1, which is known to be recruited to SSB sites^33^. We observed that EGFP-XRCC1 was rapidly recruited to the line-scanned area (Fig. 2C), indicating the production of SSBs by 405 nm laser scanning. Using cells expressing EGFP-Topo IIβ, we repeated the experiment and observed that EGFP-Topo IIβ was not recruited to the scanned area (Fig. 2C). When cells were pretreated with Hoechst 33342, EGFP-Topo IIβ was recruited to the scanned area (Fig. 2C), supporting the notion that Topo IIβ is recruited to DSB sites.

### Recruitment of Topo IIβ to DSB sites is independent of ATM and DNA-PKcs

**Figure 3:**
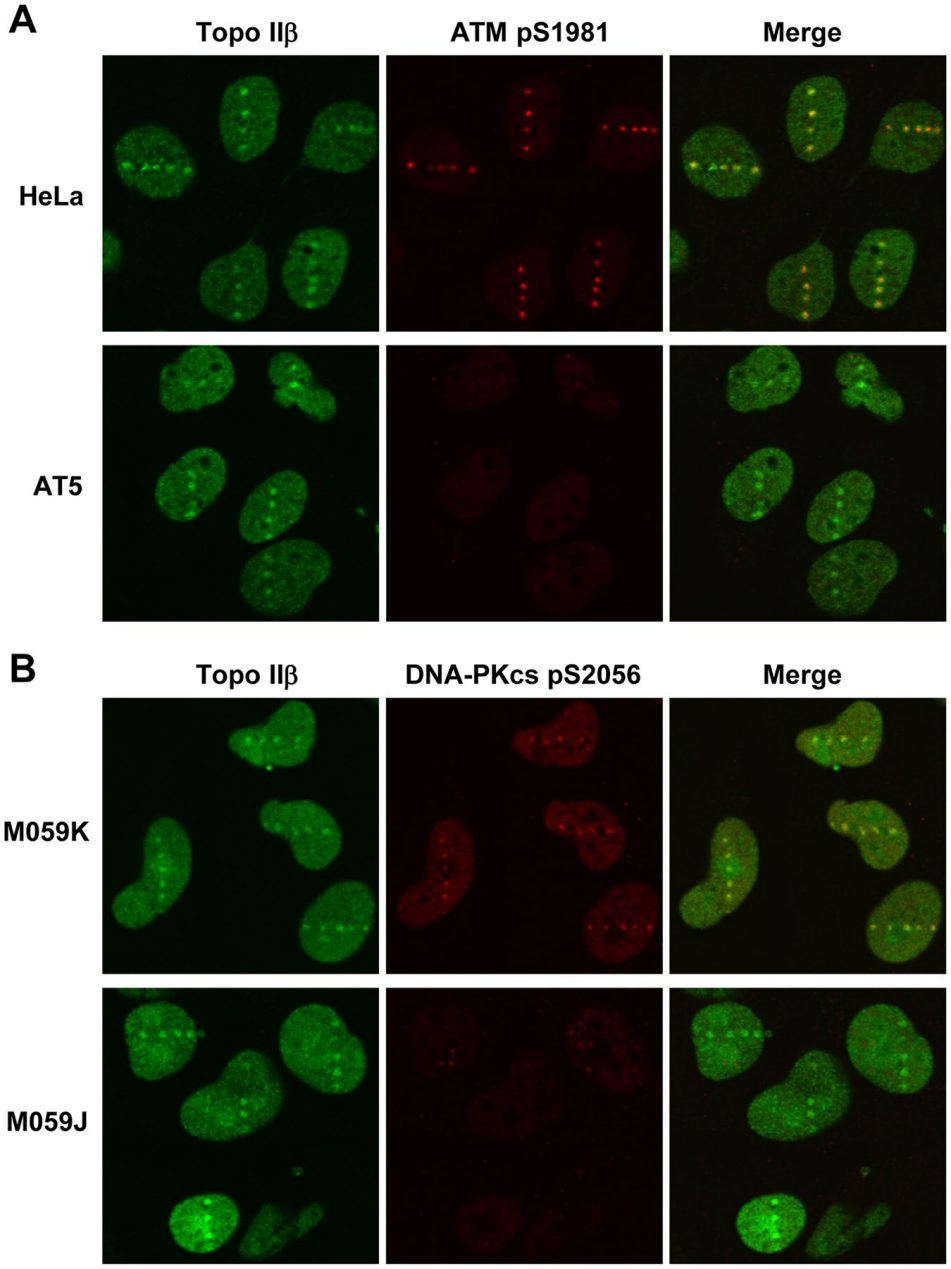
ATM- and DNA-PKcs-independent recruitment of Topo IIβ to DSB sites. (**A**) Recruitment of Topo IIβ to DSB sites in ATM-deficient cells. HeLa and ATM-deficient AT5 cells were microirradiated with a pulsed UVA laser. After 10 min incubation at 37 °C, the cells were fixed and immunostained for Topo IIβ and phosphorylated serine 1981 (pS1981) of ATM. (**B**) Recruitment of Topo IIβ to DSB sites in DNA-PKcs-deficient cells. The DNA-PKcs-proficient M059K and -deficient M059J cells were subjected to laser microirradiation followed by immunostaining for Topo IIβ and phosphorylated serine 2056 (pS2056) of DNA-PKcs.

We next investigated whether Topo IIβ recruitment to DSB sites is under the control of ATM and DNA-PKcs, both of which are protein kinases of the PI3KK family that play important roles in DSB signaling and repair^20,23^. First, we performed laser microirradiation of ATM-deficient and -proficient cells followed by coimmunostaining for endogenous Topo IIβ and phosphorylated ATM. As shown in Fig. 3A, Topo IIβ recruitment to DSB sites was indistinguishable between ATM-proficient HeLa cells and ATM-deficient AT5 cells, whereas ATM phosphorylation was detectable only in the ATM-proficient cells. We confirmed that the time-course of EGFP-Topo IIβ recruitment in ATM-deficient cells was similar to that in ATM-proficient cells (Supplementary Fig. S2). These observations indicate that ATM activation is dispensable for Topo IIβ recruitment. We next carried out a similar experiment on Topo IIβ and phosphorylated DNA-PKcs by using M059K and M059J, which are DNA-PKcs-proficient and -deficient cell lines, respectively. We observed the recruitment of Topo IIβ to the laser-induced DSB sites in both M059K and M059J cells (Fig. 3B). As shown in Supplementary Fig. S3, the time-course of EGFP-Topo IIβ recruitment to laser-damaged sites was indistinguishable between M059K and M059J cells. Collectively, these results indicate that Topo IIβ recruitment to DSB sites is not dependent on ATM or DNA-PKcs.

### Attenuation of Topo IIβ recruitment to DSB sites by inhibitors for HDAC and PARP-1

**Figure 4:**
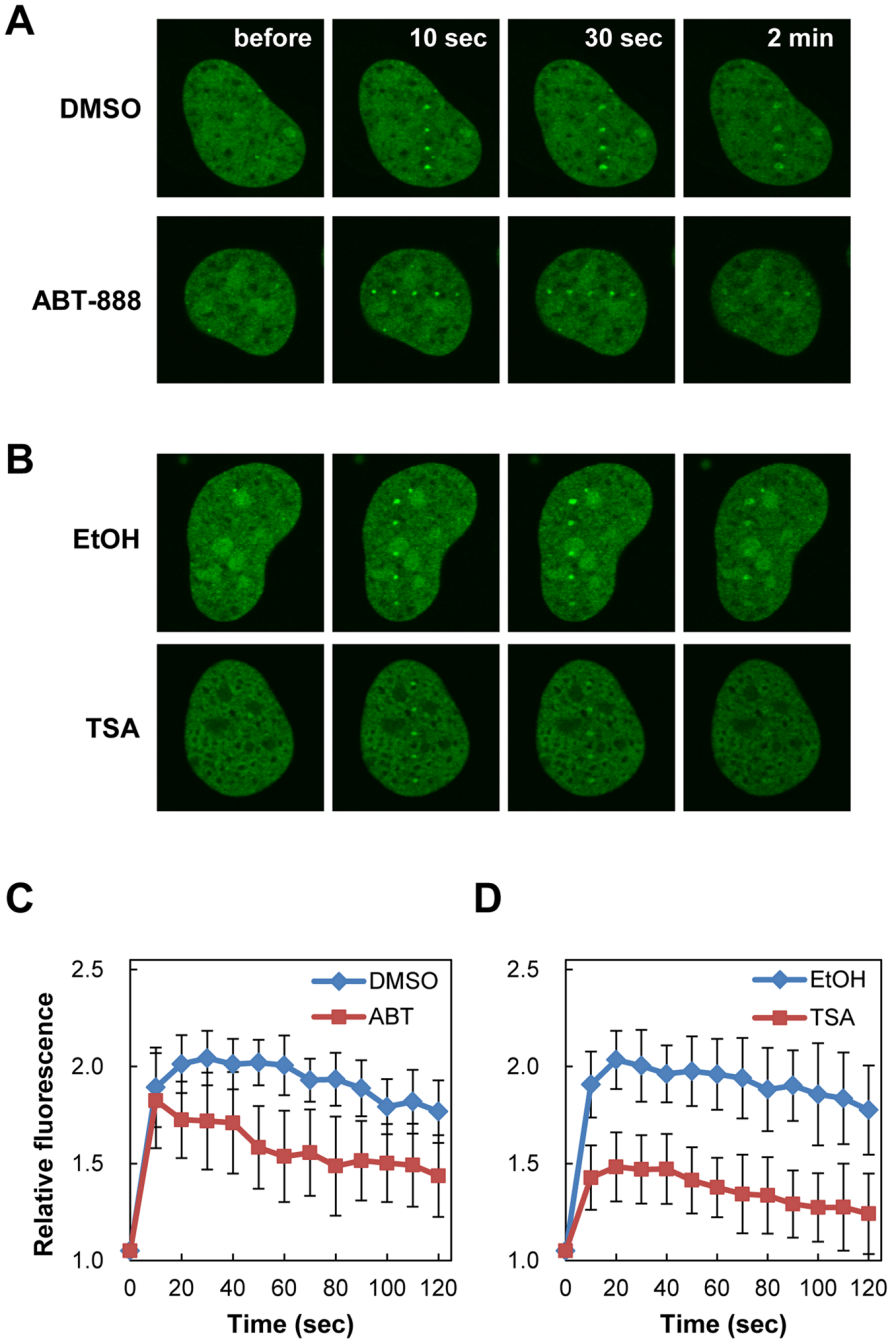
Suppression of Topo IIβ recruitment to DSB sites by inhibitors for PARP-1 and HDAC. (**A**) Recruitment of EGFP-Topo IIβ to DSB sites in the presence of ABT-888. HeLa cells expressing EGFP-Topo IIβ were pretreated with 2 µM ABT-888 or vehicle (DMSO) for 1 h and subsequently subjected to laser microirradiation. (**B**) Recruitment of EGFP-Topo IIβ to DSB sites in the presence of TSA. HeLa cells expressing EGFP-Topo IIβ were pretreated with 1 µM TSA or vehicle (ethanol) for 16 h and subsequently subjected to laser microirradiation. (**C**) Quantification of EGFP-Topo IIβ recruitment to DSB sites in the presence of ABT-888. A single shot of pulsed UVA laser was applied to the nucleus. Fluorescent images were captured at the indicated time points after laser microirradiation. EGFP signals in the DSB sites of 10 cells were quantified, and average values with SD were calculated. (**D**) Quantification of EGFP-Topo IIβ recruitment to damage sites in the presence of TSA. A single shot of pulsed UVA laser was applied to the nucleus. Fluorescent images were captured at the indicated time points after laser microirradiation. EGFP signals in the damaged sites of 10 cells were quantified, and average values with SD were calculated.

Previous studies have demonstrated that cellular responses to DSB induction are modulated by posttranslational modifications of histones and chromatin-associated proteins around DSB sites^25,26^. To gain a better understanding of Topo IIβ recruitment to DSB sites, we analyzed the effects of inhibitors of PARP-1 and HDAC, both of which play important roles in DSB-induced chromatin modifications. First, we treated HeLa cells expressing EGFP-Topo IIβ with the PARP-1 inhibitor ABT-888 and subsequently performed laser microirradiation followed by live cell imaging. Although EGFP-Topo IIβ was recruited to DSB sites in both the presence and absence of ABT-888, its foci became faint faster in the presence of ABT-888 (Fig. 4A). Quantification analysis demonstrated that the kinetics of EGFP-Topo IIβ recruitment in the presence of ABT-888 was similar with that of the vehicle control (Fig. 4C). However, retention of EGFP-Topo IIβ at DSB sites in the presence of ABT-888 was lower than that of the vehicle control (Fig. 4C). Next, we analyzed the effect of Trichostatin A (TSA), a HDAC inhibitor, on the recruitment of EGFP-Topo IIβ to DSB sites. When cells were treated with TSA prior to laser microirradiation, we observed weak fluorescent signals for EGFP-Topo IIβ foci at DSB sites (Fig. 4B). Quantification analysis confirmed that TSA treatment decreased Topo IIβ recruitment to DSB sites (Fig. 4D). Taken together, these observations indicate that the catalytic activities of PARP-1 and HDAC are required for Topo IIβ recruitment, presumably through chromatin remodeling around DSB sites.

### Suppression of Topo IIβ recruitment to DSB sites by ICRF-187 and ICRF-193

**Figure 5:**
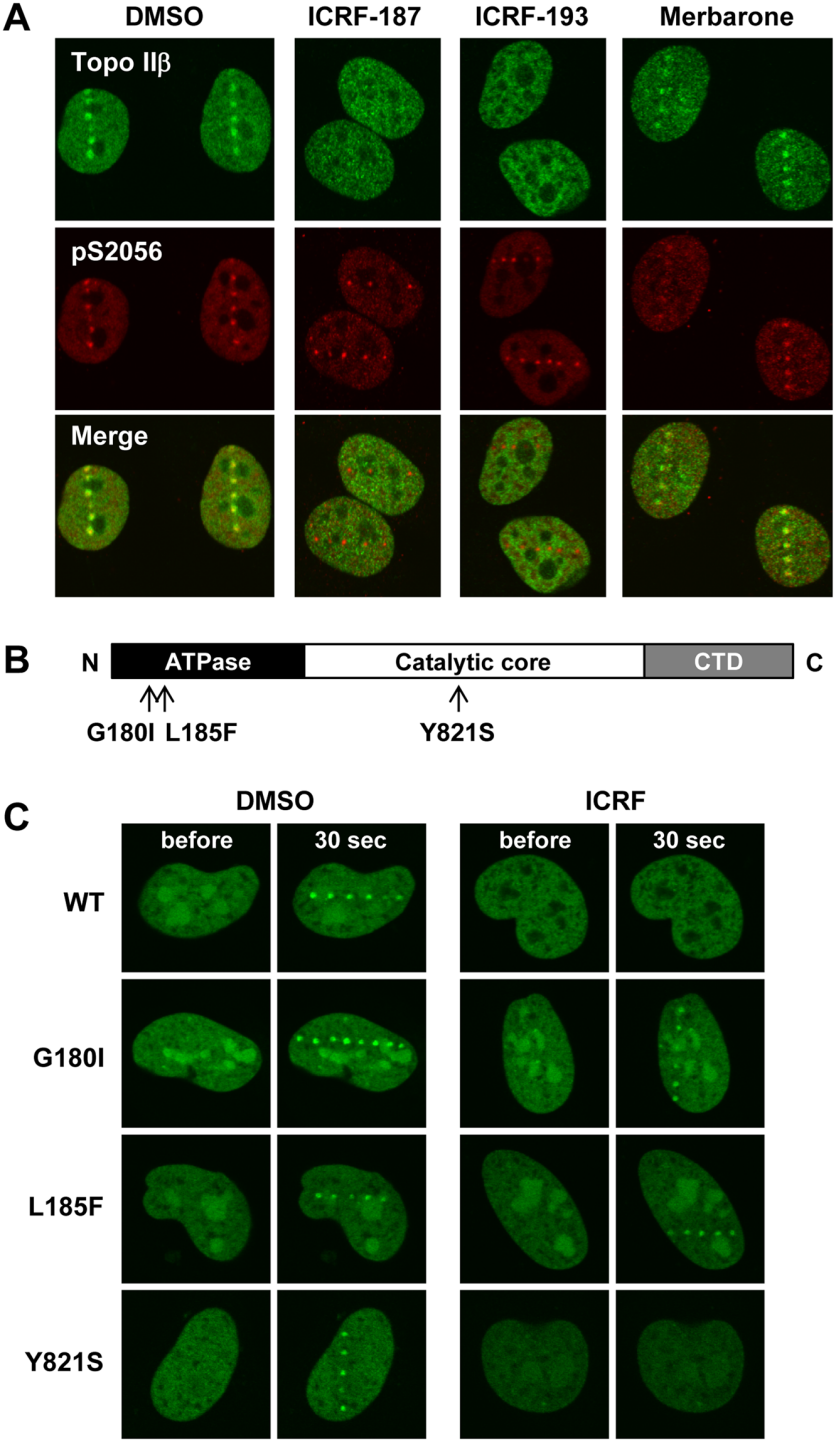
Suppression of Topo IIβ recruitment to DSB sites by ICRF-187 and ICRF-193. (**A**) Immunofluorescent staining of endogenous Topo IIβ and phosphorylated DNA-PKcs in the presence and absence of Topo II inhibitors. HeLa cells were pretreated with DMSO, 20 µM ICRF-187, 20 µM ICRF-193, or 40 µM merbarone for 1 h and subsequently subjected to laser microirradiation. After 10 min incubation at 37 °C, cells were coimmunostained for Topo IIβ and pS2056 of DNA-PKcs. Representative images are shown. (**B**) Schematic diagram of domains and amino acid substitutions in human Topo IIβ. Topo IIβ is composed of an ATPase domain, a catalytic core domain, and a C-terminal domain (CTD). The amino acid substitutions G180I and L185F are considered to confer insensitivity of Topo IIβ to the ICRF inhibitors. The Y821S substitution is inferred to impair the topoisomerase activity. (**C**) Recruitment of EGFP-Topo IIβ mutants to DSB sites in the presence of ICRF-187. EGFP-Topo IIβ mutants were transiently expressed in HeLa cells. After pretreatment with DMSO or ICRF-187 for 1 h, cells were subjected to laser microirradiation followed by live cell imaging. Representative images at 30 sec after DSB induction are shown.

The Topo II catalytic inhibitors ICRF-187 and ICRF-193 halt the conformational transitions of Topo II at the closed clamp step^5,6^. In contrast to Topo II poisons, treatment with these inhibitors does not cause a significant increase in DSBs. Using ICRF-187 and ICRF-193, we next investigated the impact of conformational restraints of Topo IIβ on recruitment to DSB sites. Cells were pretreated with either ICRF-187 or ICRF-193 and then subjected to laser microirradiation followed by coimmunostaining for endogenous Topo IIβ and phosphorylated DNA-PKcs proteins. As shown in Fig. 5A, Topo IIβ recruitment to DSB sites was completely abrogated by these inhibitors, although the foci formation of phosphorylated DNA-PKcs was unaffected. For comparison to a different class of Topo II catalytic inhibitors, we next used merbarone, which inhibits DNA cleavage by Topo II without affecting DNA-Topo II association^34^. We found that merbarone did not interfere with Topo IIβ recruitment to DSB sites (Fig. 5A).

Previous studies have reported that amino acid substitutions alter the enzymatic properties and ICRF sensitivity of Topo II^32,35–37^. Thus, we next performed experiments using Topo IIβ mutants lacking ATPase or catalytic activities. In rat Topo IIβ, a Gly-to-Ile substitution at position 173 (G173I) abolishes ATPase activity and confers resistance to ICRF-187/193^32,35^. Similarly, rat Topo IIβ harboring an L178F substitution in its ATPase domain is insensitive to the ICRF compounds^32,36^. A Y814S substitution in the catalytic core domain of rat Topo IIβ abolishes the Topo II catalytic activity^32,37^. The amino acid sequences of rat and human Topo IIβ are highly homologous, and the amino acid residues G173, L178, and Y814 in rat Topo IIβ correspond to G180, L185, and Y821 in human Topo IIβ, respectively. Assuming the functional conservation of these amino acid residues in rat and human Topo IIβ, we constructed expression plasmids for EGFP-tagged human Topo IIβ mutants (Fig. 5B) and analyzed the recruitment of these mutant proteins to DSB sites with or without ICRF-187 treatment. In the absence of ICRF-187, all mutant proteins were recruited to DSB sites in a similar manner to that seen for the wild-type protein (Fig. 5C). This indicates that neither ATPase nor Topo II catalytic activity was required for Topo IIβ recruitment to DSB sites. ICRF-187 treatment abrogated the recruitment of EGFP-tagged wild-type and Y821S Topo IIβ proteins to DSB sites, but did not affect recruitment of the G180I and L185F mutants, both of which are considered to be insensitive to ICRF inhibitors (Fig. 5C). In addition to ICRF-187, we observed similar inhibitory effects of ICRF-193 on EGFP-Topo IIβ wild-type and Y821S proteins, but not on the G180I and L185F mutants (data not shown). These observations support a causal relationship between ICRF treatment and the suppression of Topo IIβ recruitment to DSB sites.

### ICRF-187 reduces the nuclear mobility of Topo IIβ

**Figure 6:**
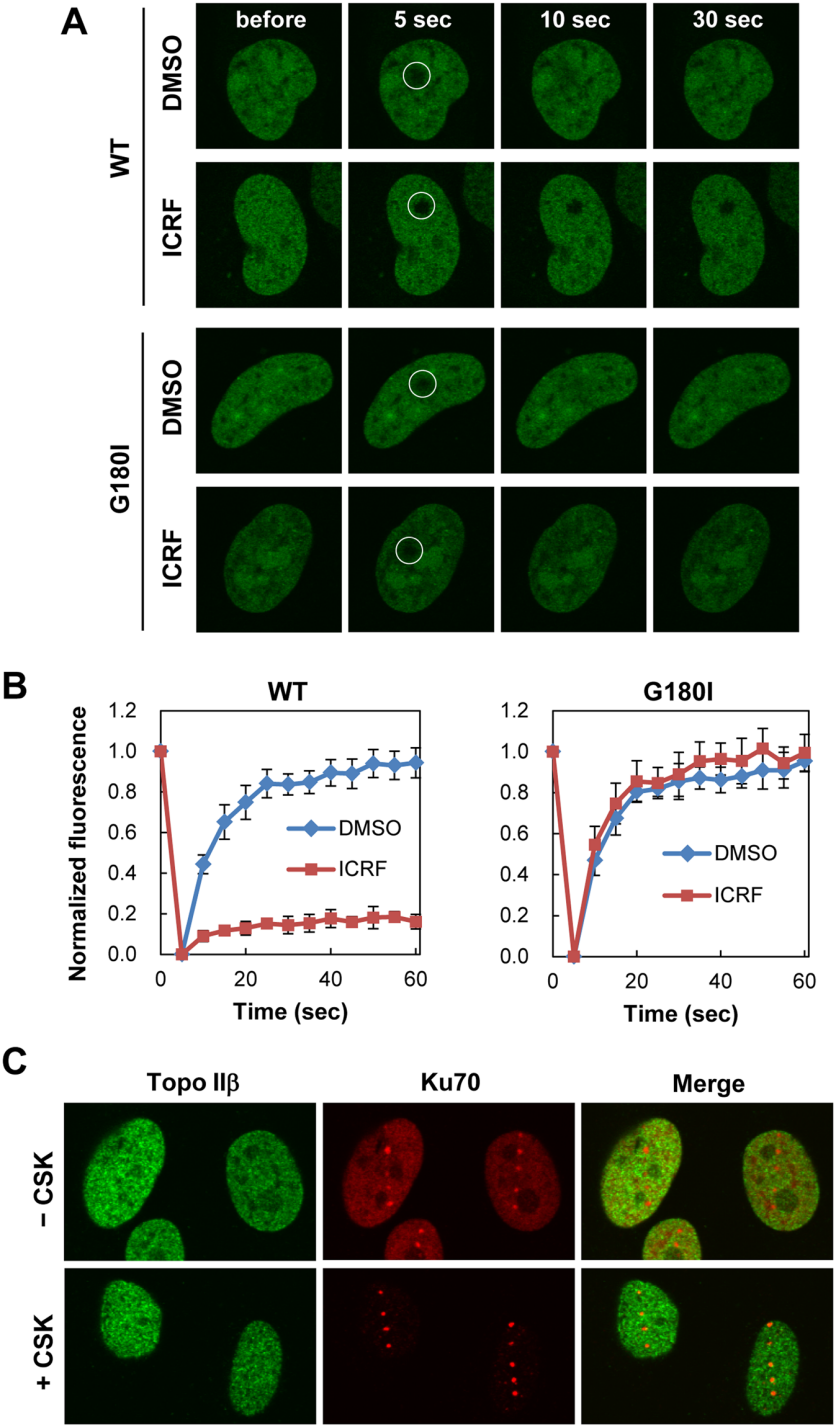
Reduced nuclear mobility of Topo IIβ by ICRF-187. (**A**) Representative images of FRAP analysis of EGFP-Topo IIβ. HeLa cells transiently expressing EGFP-Topo IIβ or EGFP-Topo IIβ carrying a G180I substitution were pretreated with either 20 µM ICRF-187 or DMSO. A small nuclear area indicated by a white circle in the nucleus was photobleached, and fluorescent images were obtained at the indicated time points. (**B**) Quantification of fluorescent signals of EGFP-Topo IIβ in FRAP analysis. Fluorescent images were obtained at 5 sec intervals. Mean values with SD from seven individual experiments were plotted. (**C**) Tight association of endogenous Topo IIβ with nuclear matrices in the presence of ICRF-187. HeLa cells pretreated with ICRF-187 were microirradiated with a pulsed UVA laser. The cells were incubated with detergent-containing (+CSK) or detergent-free (−CSK) buffer and subsequently subjected to fixation followed by coimmunofluorescence of Topo IIβ and Ku70.

A previous study on Topo IIα demonstrated that ICRF-187 decreases the nuclear mobility of Topo IIα and thereby inhibits Topo IIα functions in mitosis^38^. Based on this report, we speculated that ICRF-187 may also affect Topo IIβ mobility. To test this idea, we performed fluorescence recovery after photobleaching analysis (FRAP analysis) of EGFP-Topo IIβ in undamaged cells. Cells expressing EGFP-Topo IIβ were pretreated with ICRF-187. Fluorescence of EGFP-Topo IIβ in a small nuclear area was photobleached, and its recovery was monitored by time-lapse imaging. In the absence of ICRF-187, EGFP-Topo IIβ was highly mobile in the nucleus as evident from the rapid recovery of the EGFP signals in the photobleached area (Fig. 6A). Quantification of FRAP data demonstrated that virtually all EGFP-Topo IIβ molecules were mobile, since there was nearly 100% fluorescence recovery after photobleaching (Fig. 6B). In the presence of ICRF-187, fluorescence recovery in the photobleached area was barely detectable in fluorescence microscopy (Fig. 6A). Approximately 20% of EGFP-Topo IIβ was estimated to be mobile from quantification analysis (Fig. 6B), suggesting that a large fraction of EGFP-Topo IIβ was tethered to chromatin. The mobility of the ICRF-insensitive EGFP-Topo IIβ G180I mutant was not affected by ICRF-187 treatment, confirming causal attribution of ICRF-187 to reduced Topo IIβ mobility (Fig. 6A,B).

To investigate effect of ICRF-187 on the endogenous Topo IIβ, we performed detergent extraction of microirradiated cells followed by immunofluorescence of endogenous Topo IIβ. In the presence of ICRF-187, HeLa cells were microirradiated and incubated with or without detergent. Following fixation, cells were subjected to coimmunofluorescence of Topo IIβ and Ku70. As shown in Fig. 6C, fluorescent signals for Topo IIβ in ICRF-treated cells were distributed across the nucleus and resistant to detergent extraction. The nuclear distribution of Ku70 was not affected by ICRF-187; Ku70 was retained at DSB sites and was washed out from undamaged area after detergent treatment (Fig. 6C). Taken together with the observations from the FRAP analysis, we concluded that ICRF-187 caused a tight association of Topo IIβ with chromatin and reduced the mobility of Topo IIβ, which could account for the failure of Topo IIβ recruitment to DSB sites.

To compare with the effects of Topo II catalytic inhibitors, we next tested etoposide, a Topo II poison. As shown in Supplementary Fig. S4, the nuclear mobility of EGFP-Topo IIβ was slightly reduced by etoposide, presumably because a fraction of EGFP-Topo IIβ was tethered to chromatin DNA. A similar observation on the slightly reduced Topo IIβ mobility in etoposide-treated cells was previously reported^39^. We also analyzed the recruitment of EGFP-Topo IIβ to laser-damaged sites in etoposide-treated cells (Supplementary Fig. S5). Although we observed a slight reduction in EGFP-Topo IIβ recruitment to laser-damaged sites, the difference between etoposide-treated and untreated cells was not statistically significant, suggesting that a considerable amount of EGFP-Topo IIβ remained untethered and was sufficient to form visible accumulation at laser-damaged sites in etoposide-treated cells.

### Increased bleomycin sensitivity and reduced HR-mediated DSB repair in Topo IIβ knockout cells

**Figure 7:**
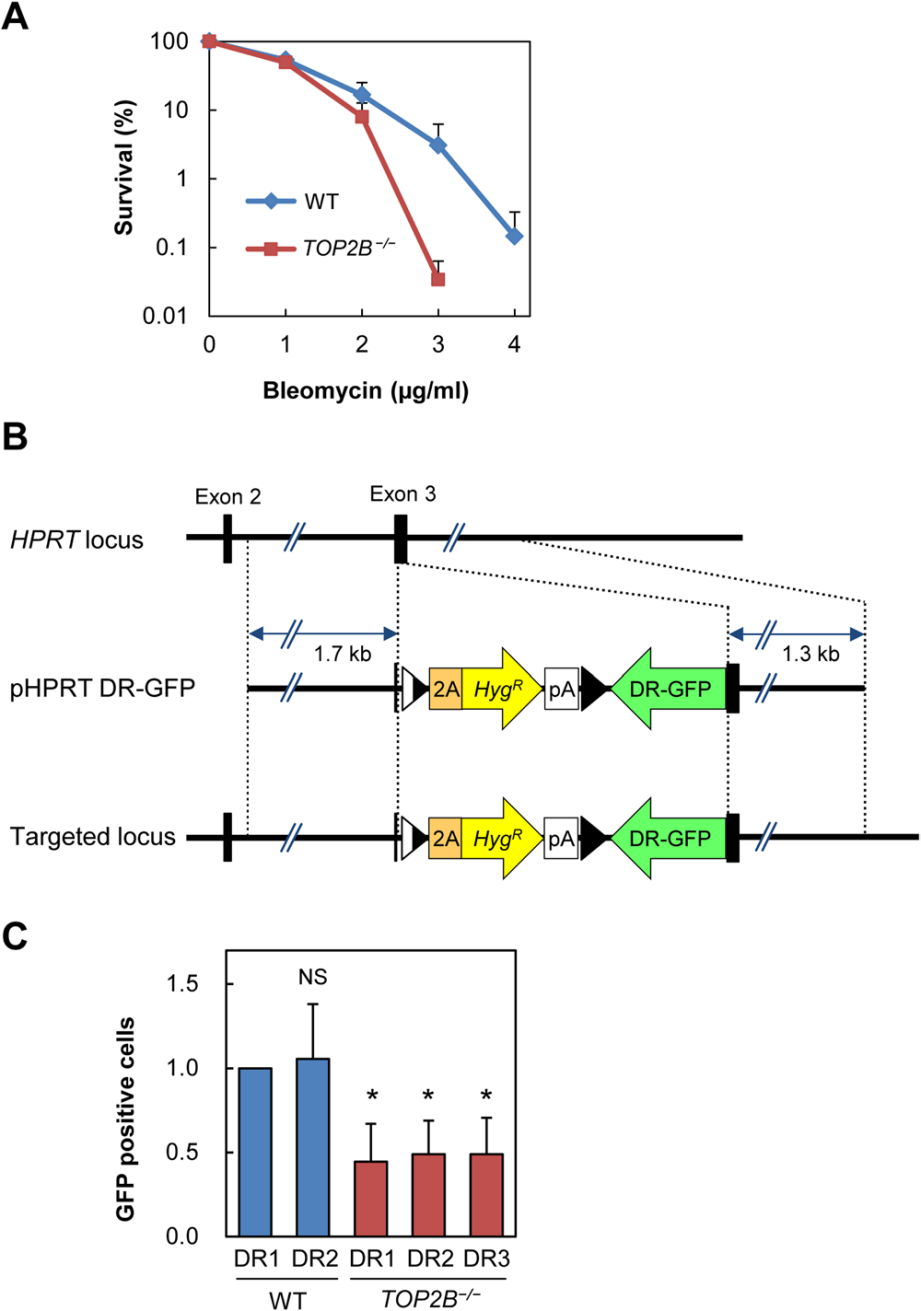
Increased bleomycin sensitivity and decreased HR-mediated DSB repair of Topo IIβ knockout cells. (**A**) Sensitivity of Topo IIβ knockout and wild-type cells to bleomycin. Clonogenic survival assays were performed using Topo IIβ knockout and wild-type (WT) Nalm-6 cells. Data are the mean ± SD of three independent experiments. Note that the vertical axis was depicted in a logarithmic scale, and the survival rates of wild-type and knockout cells at 2 μg bleomycin were 16.7% and 8.0%, respectively (p = 0.058). (**B**) Schematic representation of knock-in of the DR-GFP reporter gene into the *HPRT* locus. The pHPRT DR-GRP plasmid was knocked-in into exon 3 of the *HPRT* gene that is located on the X chromosome. Two wild-type and three Topo IIβ knockout clones were isolated. (**C**) Effect of Topo IIβ knockout on HR-mediated DSB repair. I-SceI was transiently expressed to introduce a DSB in the DR-GFP reporter gene. HR-mediated repair of the DSB in the reporter yielded GFP-positive cells that were scored by flow cytometry. DSB repair activity in a wild type clone (DR1) was set to 1. Relative DSB repair activities in another wild-type clone and three Topo IIβ-knockout clones were indicated. Data represents the mean ± SD of five independent experiments (*p < 0.05; NS, Not significant).

Given the recruitment of Topo IIβ to DSB sites, we next sought to gain insight into the role of Topo IIβ in DSB repair. We have previously generated human Topo IIβ knockout cells by gene targeting in the Nalm-6 cell line^40^. First, we analyzed the sensitivity of Topo IIβ knockout cells to the DNA damaging agent bleomycin. As shown in Fig. 7A, Topo IIβ knockout cells were more sensitive to bleomycin than wild-type cells, which was particularly evident at high doses. Because HR plays a larger role in the repair of severe DSBs than NHEJ^41^, we next examined the effect of Topo IIβ gene knockout on HR-mediated DSB repair. The DR-GFP reporter cassette for the HR assay^42^ was knocked-in to the *HPRT* locus on the X chromosome of Topo IIβ knockout and wild-type cells (Fig. 7B). We isolated two wild-type and three Topo IIβ knockout clones, each of which was confirmed to carry a single copy of the DR-GFP reporter gene on the X chromosome. The rare cutting endonuclease I-SceI was transiently expressed in these clones to generate a DSB at the I-SceI site in the DR-GFP reporter. When a DSB in the DR-GFP reporter is repaired by HR, this yields GFP-positive cells. As shown in Fig. 7C, we observed that the proportions of GFP-positive cells in three Topo IIβ knockout clones were approximately 50% of the wild-type clones, indicating that Topo IIβ knockout cells display reduced HR activity for DSB repair. As shown in Supplementary Fig. S6, we performed immunostaining of Rad51 in wild-type and Topo IIβ-knockout cells after NCS treatment and observed that there was no significant difference of Rad51 focus formation between these cells. Furthermore, the distribution of cell cycle stages was essentially indistinguishable between wild type and Topo IIβ-knockout cells (data not shown), which is in accord with the previously published observation^43^.

## Discussion

Topo IIβ has attracted considerable attention for its versatile biological functions and clinical importance as a target of anti-tumor drugs. In contrast to the increasing knowledge regarding the biological and clinical relevance of Topo IIβ, limited information is available on the dynamic behavior of Topo IIβ, particularly in relation to DSB induction. In this study, we investigated the response of Topo IIβ to DSB induction and its physiological significance. Live cell-imaging showed rapid and transient recruitment of EGFP-Topo IIβ to DSB sites (Fig. 2A), and FRAP analysis demonstrated the high mobility of EGFP-Topo IIβ in the nucleus (Fig. 6A,B). The Topo II catalytic inhibitors ICRF-187 and ICRF-193 decreased the nuclear mobility of EGFP-Topo IIβ and prevented the recruitment of EGFP-Topo IIβ to DSB sites (Figs 5,6). In accord with the observation on EGFP-Topo IIβ, ICRF-187 treatment markedly increased the residence of endogenous Topo IIβ in the detergent-extracted nucleus (Fig. 6C). These observations indicate that high mobility in the nucleus ensures the rapid response of Topo IIβ to DSB induction. Since ICRF-187 and ICRF-193 are known to trap the homodimeric Topo II protein as a closed clamp on a DNA strand^5,6^, we speculated that these inhibitors caused the tethering of Topo IIβ to chromatin DNA and thereby interfered with the translocation of Topo IIβ to DSB sites.

We observed that NCS treatment did not yield clear foci of Topo IIβ (Supplementary Fig. S1). We speculate that Topo IIβ is recruited to DSBs at relatively low abundance as is the case for Ku^44,45^ and other proteins^45–48^. Previous studies have demonstrated that visible foci do not necessarily reflect the initial recruitment step of DSB responses. Rather, subsequent signal amplification and protein clustering surrounding the damaged sites are more important for the formation of visible foci^44,49^. In the case of Ku, which is well known to tightly bind to DSBs, ionizing radiation (IR) does not cause accumulation of Ku at sufficient levels to be visible as foci^50–52^. Laser microirradiation can induce DSBs at a high density in a small focused area and thus enables cytological observation of the recruitment of factors that do not form visible foci by IR^28,44,53,54^. In addition to the low abundance of recruited Topo IIβ, we speculate that fast kinetics of Topo IIβ recruitment also hampers the detection of Topo IIβ foci in NCS-treated cells. Given the fast kinetics of EGFP-Topo IIβ association/dissociation at damaged sites (Fig. 2), we suppose that NCS treatment followed by fixation and immunostaining requires too much time for the detection of Topo IIβ foci. Collectively, we speculate that Topo IIβ binds to DSBs at relatively low abundance and with fast kinetics.

Our findings in this study raise new questions that need to be addressed in future research. First, the mechanism for Topo IIβ recruitment to damaged sites remains an open question. Among various forms of DNA damage, we inferred that Topo IIβ is recruited to DSBs, because the recruitment of Topo IIβ to 405 nm laser scanning sites required presensitization with Hoechst 33342 (Fig. 2C). Based on this notion, we examined the possible participation of two major regulatory factors, namely ATM and DNA-PKcs, in Topo IIβ recruitment. Although ATM deficiency frequently causes various defects in cellular responses to DSB induction, Topo IIβ recruitment to DSB sites was indistinguishable between ATM-proficient and -deficient cells (Fig. 3A). DNA-PKcs plays important roles in NHEJ-mediated DSB repair and other cellular activities. Notably, previous studies have shown that Topo IIβ associates with DNA-PKcs on the promoter regions of several hormone-responsive genes^16–18^. Although the association between Topo IIβ and DNA-PKcs is crucial for transcriptional control^17^, this was not the case for the recruitment of Topo IIβ to DSB sites, since Topo IIβ recruitment was observed in cells lacking DNA-PKcs (Fig. 3B). Furthermore, although merbarone was reported to disrupt the association of Topo IIβ with DNA-PKcs and abrogate glucocorticoid-induced transcription^18^, it did not affect the recruitment of Topo IIβ to DSBs (Fig. 5A). Taken together, these observations demonstrated that Topo IIβ recruitment to DSBs is independent of ATM or DNA-PKcs. In contrast, Topo IIβ recruitment was affected with inhibitors for PARP-1 and HDAC (Fig. 4), both of which contribute to alterations in chromatin structure around DSB sites. This suggests that Topo IIβ associates with structural alterations in local chromatin and/or modifications of chromatin proteins arising around DSBs, which deserves further investigation. Future efforts should focus on identifying a molecular event that is required for Topo IIβ recruitment upon DSB induction.

As shown in Fig. 7A, we observed that the increased sensitivity of Topo IIβ knockout cells to bleomycin was evident at higher doses. In our previous study^41^, we demonstrated that HR-deficient and NHEJ-deficient cells have similar sensitivities to a radiomimetic agent at low doses, but HR-deficient cells exhibit higher sensitivity than NHEJ-deficient ones at high doses. We had interpreted this observation as indicating that HR plays a larger role in the repair of severe DSBs than NHEJ. In line with this notion, another study proposed that DSB repair is initially undertaken by NHEJ, and when the repair is insufficient, the role of HR in DSB repair becomes more significant^55^. We thus favor the view that the greater sensitivity of Topo IIβ knockout cells to high-dose bleomycin implies the role of Topo IIβ in HR-mediated DSB repair. This idea will be validated by detailed mechanistic analysis in the future.

Our observation on the reduced activity of HR-mediated DSB repair in Topo IIβ-deficient cells led to another open question of how Topo IIβ is involved in this process. In general, Topo IIβ is not regarded as a core component of HR. In accordance with this view, we observed that Topo IIβ knockout causes a relatively modest decrease in HR-mediated DSB repair as compared with Rad51 or BRCA2 knockdown^56,57^. We hypothesize an auxiliary role of Topo IIβ in HR, presumably through indirect mechanisms, rather than direct involvement in HR. For example, Topo IIβ may have a facilitative role in the modulation of topological constraints of DNA strands imposed during the process of HR. Another possibility is a protective role of Topo IIβ in DNA topology around DSBs to ensure efficient DSB repair. We speculate that Topo IIβ deficiency may cause certain structural alterations around DSB sites and ultimately manifest as a reduced HR activity. Further detailed investigation will follow in the future, and the assessment of the impact of Topo IIβ deficiency on NHEJ in comparison to HR is also important for understanding the significance of Topo IIβ in DSB repair. In summary, the present study sheds light on novel aspects of Topo IIβ function and provides a basis for further research into the mechanism by which Topo IIβ knockout affects HR-mediated DSB repair. Future efforts will be directed toward a better understanding of the physiological significance of Topo IIβ recruitment to DSB sites.

## Methods

### Reagents

ICRF-187 and ABT-888 were obtained from Cayman Chemical (Ann Arbor, MI, USA). ICRF-193 and merbarone were purchased from Sigma-Aldrich (St. Louis, MO, USA). TSA and bleomycin were obtained from Wako Pure Chemicals (Osaka, Japan). The following antibodies were used in this study: a mouse monoclonal anti-Topo IIβ antibody (#611492, BD Biosciences, Franklin Lakes, NJ, USA), a rabbit polyclonal anti-DNA-PKcs antibody (phospho-S2056, ab18192, Abcam, Cambridge, UK), a rabbit monoclonal anti-Ku70 antibody (#4588, Cell Signaling Technology, Danvers, MA, USA), a rabbit monoclonal anti-ATM antibody (phospho-S1981, ab81292, Abcam), goat anti-mouse IgG-Alexa Fluor488 and goat anti-rabbit IgG-Alexa Fluor594 antibodies (A11029 and A11037, respectively, Thermo Fisher Scientific, Waltham, MA, USA).

### Plasmids

Full-length human Topo IIβ cDNA was cloned by The Institute of Physical and Chemical Research (RIKEN, Wako, Japan, GenBank accession number BC156330.1). Under license agreement with RIKEN, this cDNA clone was obtained from K.K. DNAFORM (Yokohama, Japan, Clone ID: 10061836). The full-length human Topo IIβ cDNA was inserted into the pCD3F2-EGFP plasmid to produce an expression plasmid encoding EGFP-Topo IIβ. Site-directed mutagenesis of the EGFP-Topo IIβ expression plasmid was performed using specific oligonucleotides to yield G180I, L185F, and Y821S substitutions. Sequence information on the oligonucleotides used for site-directed mutagenesis is available upon request. Integrity of all plasmids was verified by DNA sequencing. The pCD3F2-EGFP plasmid and the EGFP-XRCC1 expression plasmid were gifts from Prof. David Chen (University of Texas Southwestern Medical Center at Dallas).

### Cell culture and transfection

HeLa cells were obtained from RIKEN (Wako, Japan). AT5, M059K, and M059J cells were gifts from Prof. David Chen. Cells were grown in α-modified minimum essential medium (αMEM, Wako Pure Chemical) supplemented with 10% fetal bovine serum (FBS, Corning, NY, USA), 100 µg/mL streptomycin, and 100 units/mL penicillin. Wild-type and Topo IIβ knockout Nalm-6 cell lines^40^ were grown in RPMI-1640 medium (Wako Pure Chemical) supplemented with 10% FBS and penicillin/streptomycin mixture. Cells were cultured under standard conditions at 37 °C in a humidified incubator containing 5% CO_2_. Transfection of HeLa cells with an expression plasmid was performed using a FuGENE6 reagent (Promega, Madison, WI, USA) according to the manufacturer’s instructions.

### Microscopy and laser microirradiation

Microscopy was performed using an FV1200-IX83 laser scanning confocal microscope (Olympus, Tokyo, Japan). For live cell imaging, cells grown on a glass bottomed dish were placed on a stage top incubator (Tokai Hit, Shizuoka, Japan) that maintained a humidified atmosphere of 5% CO_2_ at 37 °C. Images were analyzed using FLUOVIEW software (Olympus). The pulsed UV laser at 349 nm was generated using an Explorer 349NM laser system (Spectra-Physics, Santa Clara, CA, USA), introduced into the epifluorescence path of the microscope, and focused through an oil-immersed 60× objective. The laser output and ND filter were set to 70% and 5%, respectively, which achieved delivery of approximately 3.75 µJ of energy to a punctate focus. Similar energy conditions (365 nm pulsed laser, 4 μJ) were previously reported for DSB induction without sensitizer treatment^28^. Fluorescence intensities of the irradiated region in the nucleus were measured by using FLUOVIEW software and normalized to a non-irradiated region. For SSB generation, cells were scanned with a 405 nm laser for 2 sec at 95% output. To induce DSBs under the same laser scan conditions, cells were sensitized with 0.2 µg/ml Hoechst 33342 (Dojindo Laboratories, Kumamoto, Japan) for 5 min prior to irradiation.

### FRAP analysis

HeLa cells were transiently transfected with the EGFP-Topo IIβ expression plasmid. At 24 h after transfection, a spot in the nucleoplasm was photobleached with a 473 nm laser at 35% output for 1 sec. Before and after photobleaching, fluorescent images were acquired at 5 sec intervals. Fluorescence intensity of the photobleached region was quantified using FLUOVIEW software, and fluorescence recovery was expressed as a ratio of fluorescence before and after photobleaching.

### Immunofluorescence staining

Cells were fixed with 4% paraformaldehyde (PFA) dissolved in Dulbecco’s phosphate-buffered saline (D-PBS) for 30 min at 4 °C. After washing with D-PBS, cells were permeabilized with 0.1% Triton X-100 in D-PBS for 3 min, subsequently blocked with 1% bovine serum albumin in D-PBS for 15 min, and reacted with appropriate primary antibodies. After washing with D-PBS, cells were incubated with fluorescent secondary antibodies and subsequently mounted in a Vectashield mounting medium (Vector Laboratories, Burlingame, CA, USA).

Extraction of soluble proteins prior to fixation was performed according to previously described procedures^31^ with slight modifications. Cells were incubated twice for 3 min at room temperature with cytoskeleton buffer (CSK) containing 10 mM Pipes, pH 7.0, 100 mM NaCl, 300 mM sucrose, 3 mM MgCl_2_ and 0.5% Triton X-100. After washing with D-PBS, cells were fixed with 4% PFA and subsequently subjected to immunofluorescence staining as described above.

### Drug sensitivity assay

Clonogenic survival assays using Nalm-6 and its derivatives were performed as described previously^58^. Briefly, exponentially growing cells were plated at 10^2^–10^6^ cells/dish into 60-mm dishes containing 5 ml of agarose medium with various concentrations of bleomycin. After 2–3 week incubation at 37 °C, visible colonies were counted, and the percent survival was determined by comparing the number of surviving colonies to untreated controls.

### GFP reporter assay

To compare HR frequencies between wild-type and mutant cells, we knocked in a DR-GFP reporter cassette at exon 3 of the *HPRT* locus^42^ (Fig. 7B). Construction of the targeting vector pHPRT DR-GFP will be described elsewhere. pHPRT DR-GFP was linearized with SwaI and transfected into Nalm-6 wild-type and mutant cells as described^58^. The cells were then selected doubly with 0.4 mg/ml hygromycin B and 20 µM 6-thioguanine, and the targeted integration of the reporter construct was screened by PCR using primers HPRT-F (5′-TGAGGGCAAAGGATGTGTTACGTG-3′) and HPRT-R (5′-TTGATGTAATCCAGCAGGTCAGCA-3′). The resulting targeted cell lines WT-DR1, WT-DR2, TOP2B^−/−^DR1, TOP2B^−/−^DR2, and TOP2B^−/−^−DR3, were used to assess HR frequency at the chromosomal HPRT locus. Each cell line was transfected with 2 µg of the I-SceI expression vector pSceI^59^ or pmaxGFP (Lonza, Basel, Switzerland) using the Nucleofector II system (Lonza), and cultured for 72 h. GFP-positive cells were enumerated using a JSAN cell sorter (Bay Bioscience, Kobe, Japan). For each experiment, 10^5^ cells were analyzed, and the HR frequency was calculated from the number of GFP-positive cells divided by the number of cells analyzed after correction for transfection efficiency.

## Electronic supplementary material

Supplementary Information
