# Supplementary material for: Dynamic behavior of DNA topoisomerase IIβ in response to DNA double-strand breaks
Source: Sci Rep. 2018 Jul 9;8:10344. doi: 10.1038/s41598-018-28690-6 (PMC6037730; doi:10.1038/s41598-018-28690-6)
Supplement: Supplementary file 1 — Supplementary Information [file 41598_2018_28690_MOESM1_ESM.pdf]

Supplementary Information

Dynamic behavior of DNA topoisomerase IIβ in response to DNA double-strand breaks

Keiko Morotomi-Yano[1], Shinta Saito[2], Noritaka Adachi[2, 3], Ken-ichi Yano[1, *]

[1] Department of Bioelectrics, Institute of Pulsed Power Science, Kumamoto University, Kumamoto 860-8555, Japan

[2] Department of Life and Environmental System Science, Graduate School of Nanobioscience, Yokohama City University, Yokohama 236-0027, Japan

[3] Advanced Medical Research Center, Yokohama City University, Yokohama 236-0004, Japan

* Correspondence should be addressed to K. Y. (e-mail: yanoken@kumamoto-u.ac.jp)

Supplementary Methods

NCS treatment and immunofluorescent analysis of Nalm-6 cells

Neocarzinostatin (NCS) was purchased from Sigma-Aldrich (St. Louis, MO, USA). Wild-type and Topo IIβ knockout Nalm-6 cells were suspended in RPMI medium containing 10% fetal bovine serum (FBS) and treated with and without 250 ng/ml NCS at 37°C for 2 h. Cells were collected by brief centrifugation and resuspended in RPMI medium without FBS. Cell suspension was incubated on a chamber slide at 37°C for 30 min to allow cell attachment. Attached cells were fixed with 4% paraformaldehyde dissolved in Dulbecco's phosphate-buffered saline (D-PBS) for 30 min at 4°C. After washing with D-PBS, cells were permeabilized with 0.1% Triton X-100 in D-PBS for 3 min, subsequently blocked with 1% bovine serum albumin in D-PBS for 15 min, and reacted with anti-Rad51 antibody (GTX100469, GeneTex, Irvine, CA, USA). After washing with D-PBS, cells were incubated with fluorescent secondary antibodies and subsequently mounted in a Vectashield mounting medium containing 4',6-diamidino-2-phenylindole (DAPI) (Vector Laboratories, Burlingame, CA, USA).

Figure S1

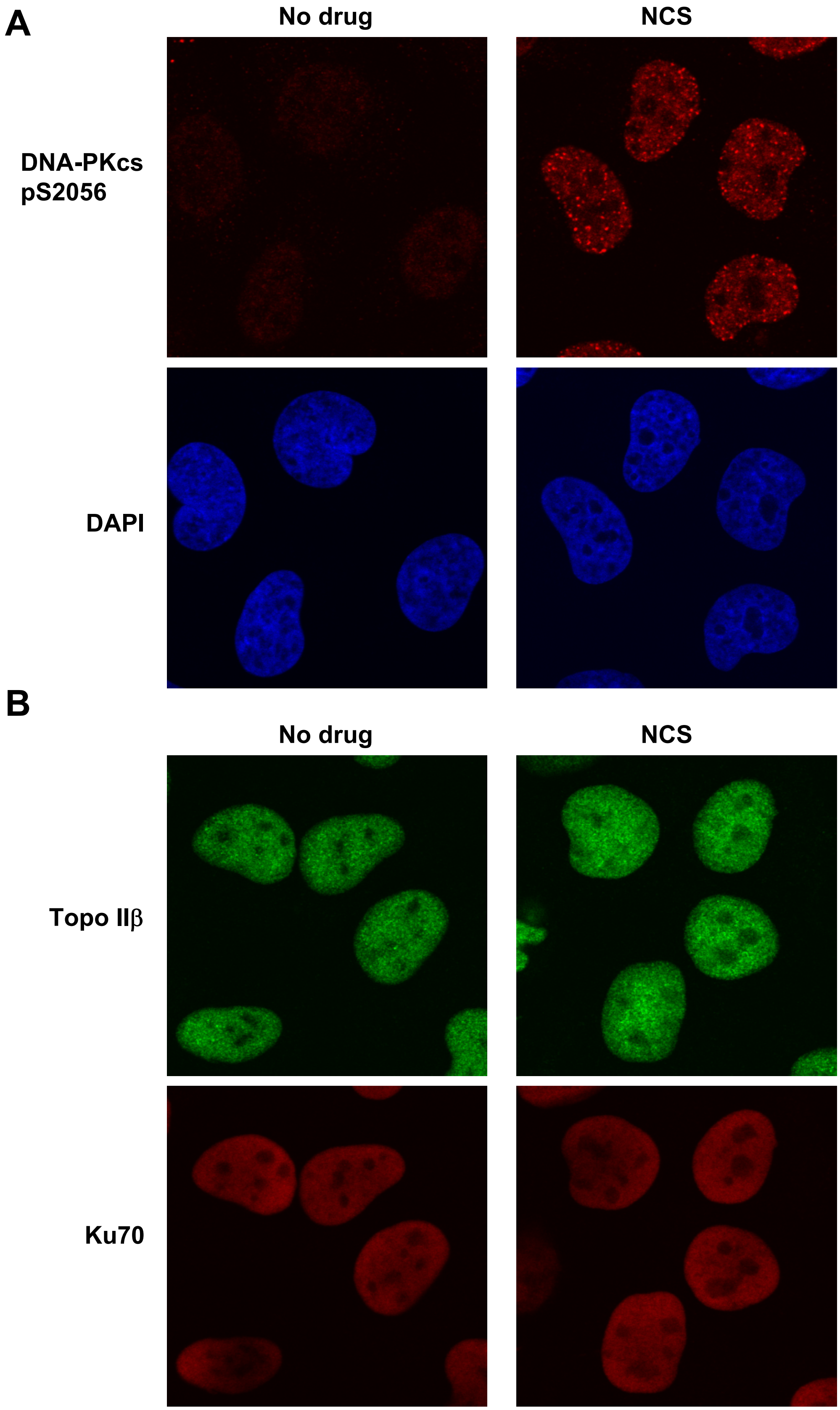

Supplementary Figure S1 Immunofluorescent analysis of Topo IIβ and Ku in cells treated with neocarzinostatin. A. HeLa cells were incubated with and without 250 ng/ml neocarzinostatin (NCS) at 37°C for 2 h and subsequently subjected to immunofluorescent staining of phosphorylated DNA-PKcs at S2056 (pS2056). Nuclear DNA was stained with DAPI. B. HeLa cells were incubated with and without NCS as described in A and subsequently subjected to coimmunofluorescent staining of Topo IIβ and Ku70.

Figure S2

A

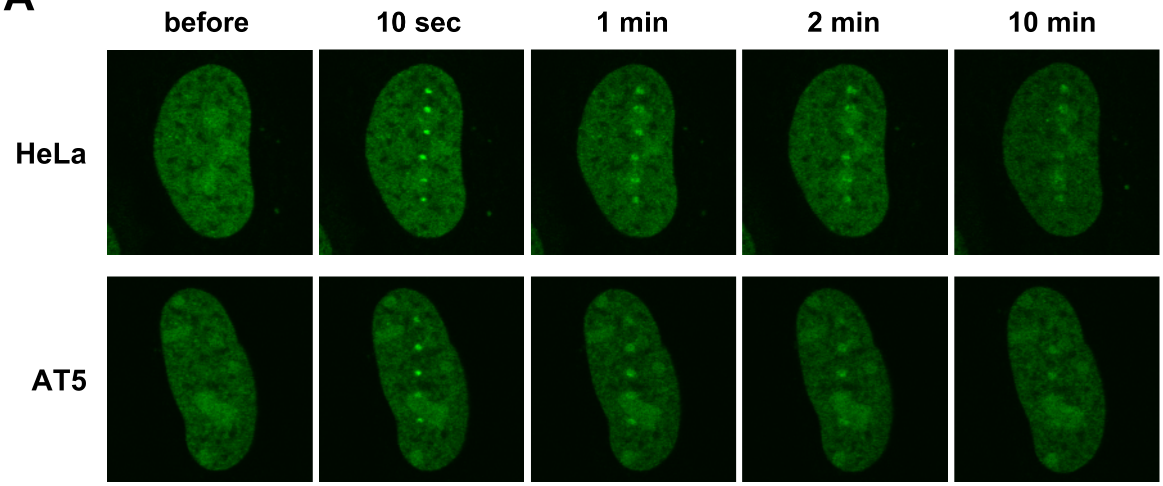

B

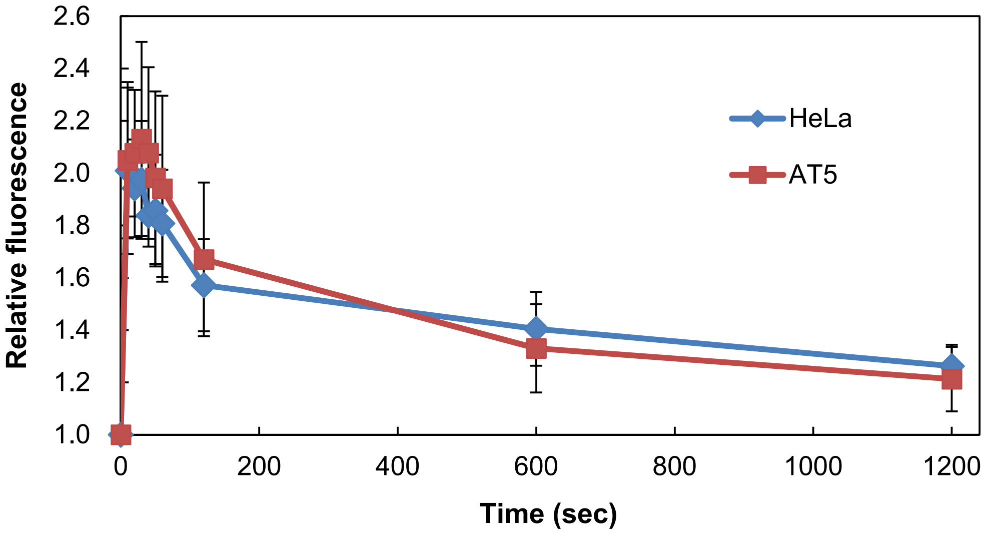

Supplementary Figure S2 Live imaging of Topo IIβ recruitment to laser-damaged sites in ATM-proficient and -deficient cells. A. EGFP-Topo IIβ was transiently expressed in HeLa and ATM-deficient AT5 cells. DNA damage was induced with shots of a pulsed UVA laser, and recruitment of EGFP-Topo IIβ to the damaged sites was monitored by time-lapse microscopy. Representative images at the indicated time points are shown. B. Time-course of EGFP-Topo IIβ recruitment to laser-damaged sites in AT5 and HeLa cells. DNA damage was induced in the nucleus with a single shot of a pulsed UVA laser. After time-lapse imaging, EGFP signals in the damaged site were quantified, and average values with SD were calculated from 10 cells.

Figure S3

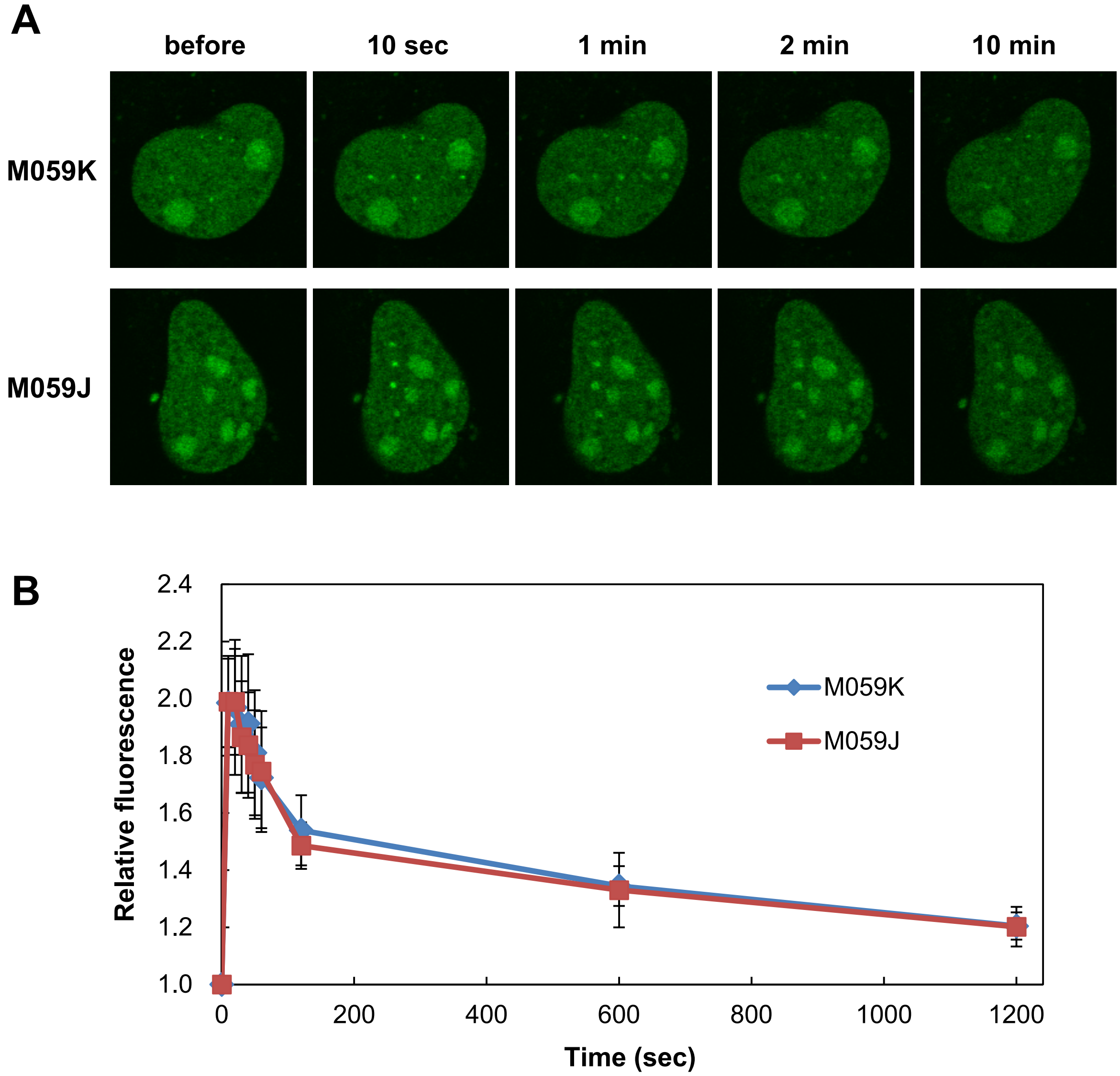

Supplementary Figure S3 Live imaging of Topo IIβ recruitment to laser-damaged sites in DNA-PKcs-proficient and -deficient cells. A. EGFP-Topo IIβ was transiently expressed in the DNA-PKcs-proficient M059K and -deficient M059J cells. DNA damage was induced with shots of a pulsed UVA laser, and recruitment of EGFP-Topo IIβ to the damaged sites was monitored by time-lapse microscopy. Representative images at the indicated time points are shown.
B. Time-course of EGFP-Topo IIβ recruitment to laser-damaged sites in M059K and M059J cells. DNA damage was induced in the nucleus with a single shot of a pulsed UVA laser. After time-lapse imaging, EGFP signals in the damaged site were quantified, and average values with SD were calculated from 10 cells.

Figure S4

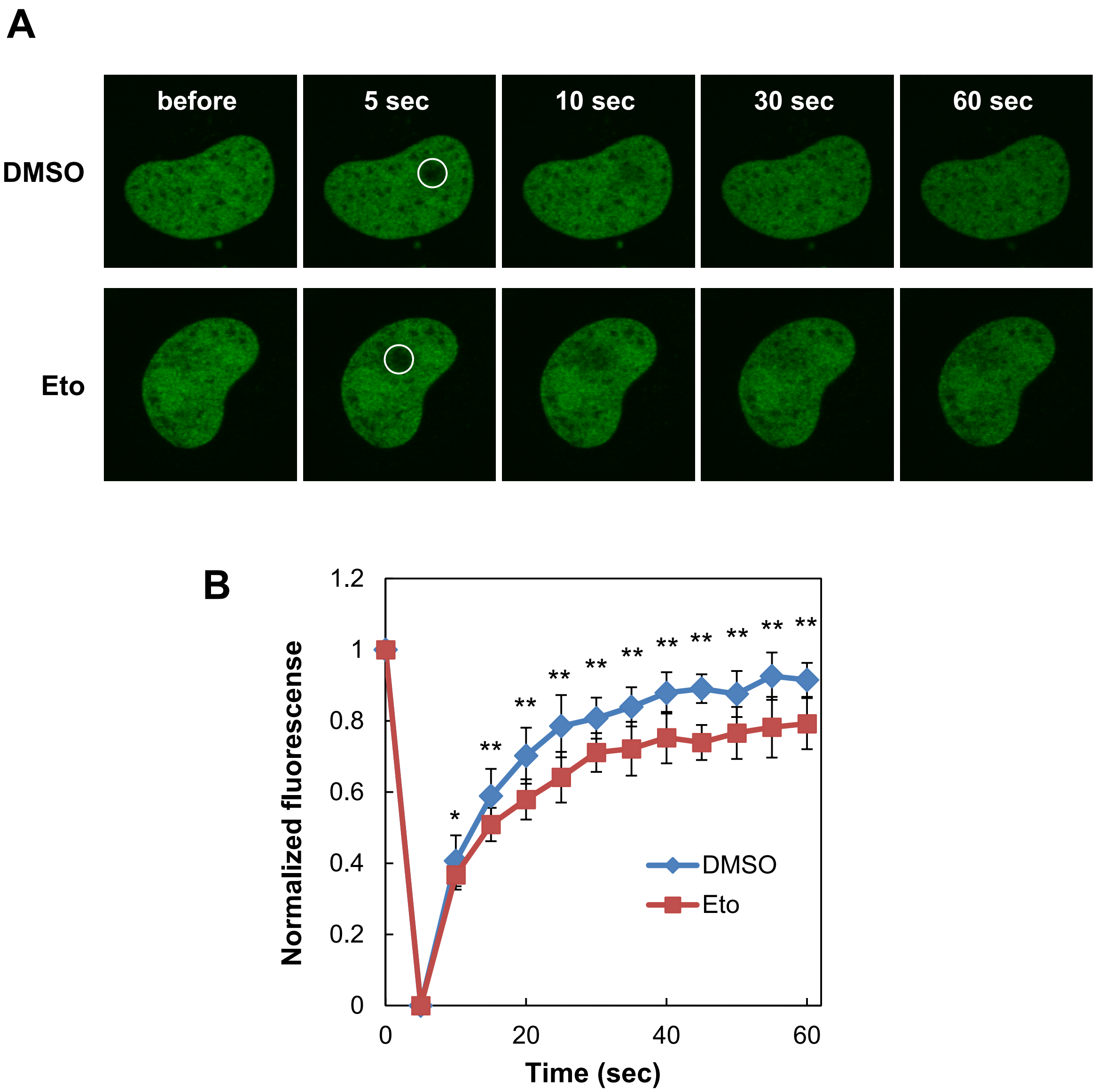

Supplementary Figure S4 Nuclear mobility of EGFP-Topo IIβ in etoposide-treated cells. A. Representative images of FRAP analysis in the presence and absence of etoposide. HeLa cells expressing EGFP-Topo IIβ were pretreated with either 25 μM etoposide (Eto) or DMSO for 2 h. A small nuclear area indicated by a white circle in the nucleus was photobleached, and fluorescent images were obtained at the indicated time points. B. Quantification of fluorescent signals of EGFP-Topo IIβ in FRAP analysis. Fluorescent images were obtained at 5 sec intervals. Mean values with SD from 10 cells were plotted (*, $p < 0.05$; **, $p < 0.01$).

Figure S5

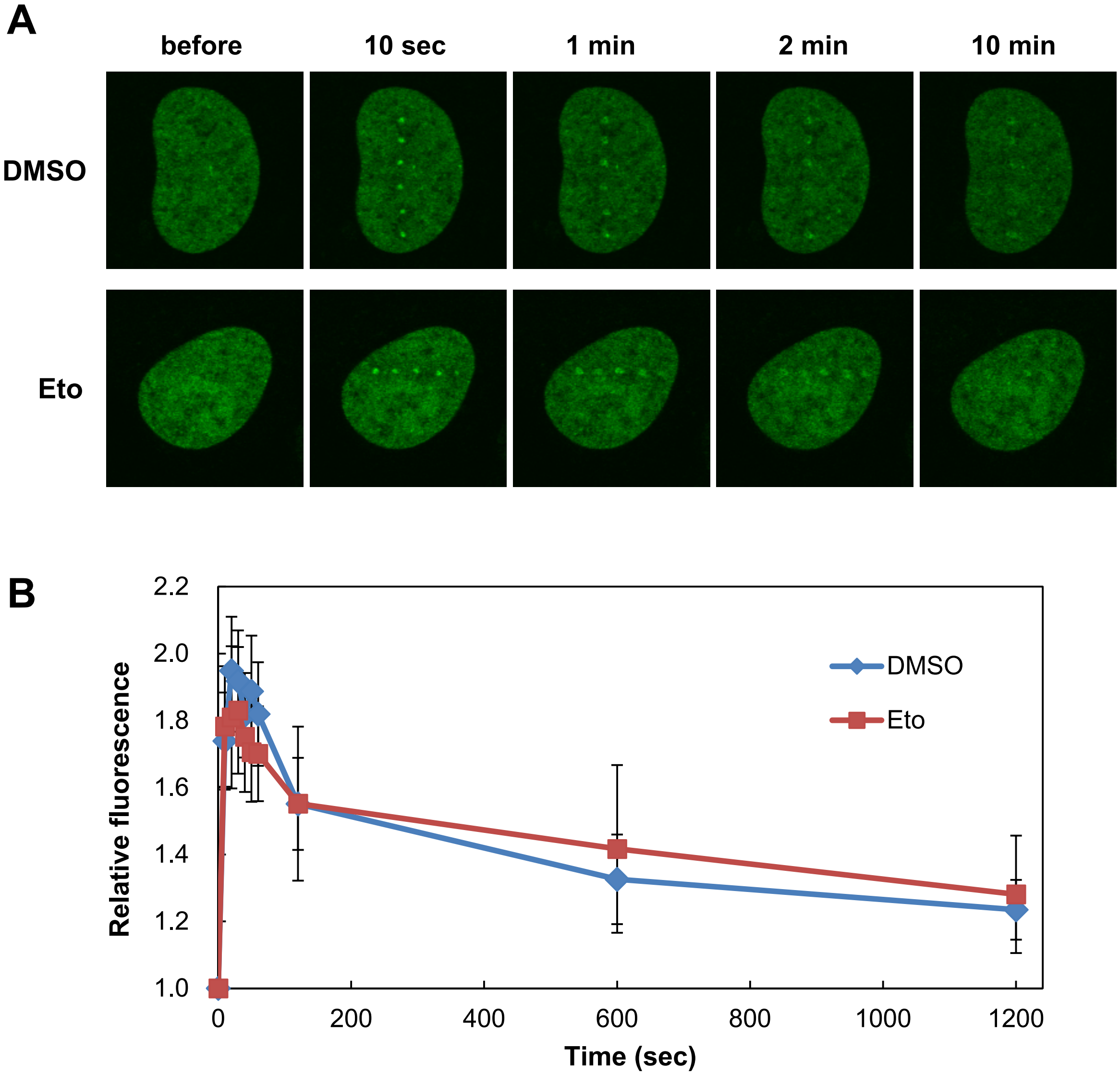

Supplementary Figure S5 Recruitment of EGFP-Topo IIβ to laser-damaged sites in etoposide-treated cells. A. Recruitment of EGFP-Topo IIβ to laser-damaged sites in the presence and absence of etoposide. HeLa cells expressing EGFP-Topo IIβ were pretreated with either 25 μM etoposide (Eto) or DMSO for 2 h. DNA damage was induced with shots of a pulsed UVA laser, and recruitment of EGFP-Topo IIβ to the damaged sites was monitored by time-lapse microscopy. Representative images at the indicated time points are shown.
B. Time-course of EGFP-Topo β recruitment to laser-damaged sites in the presence and absence of etoposide. DNA damage was induced in the nucleus with a single shot of a pulsed UVA laser. After time-lapse imaging, EGFP signals in the damaged site were quantified, and average values with SD were calculated from 10 cells.

Figure S6

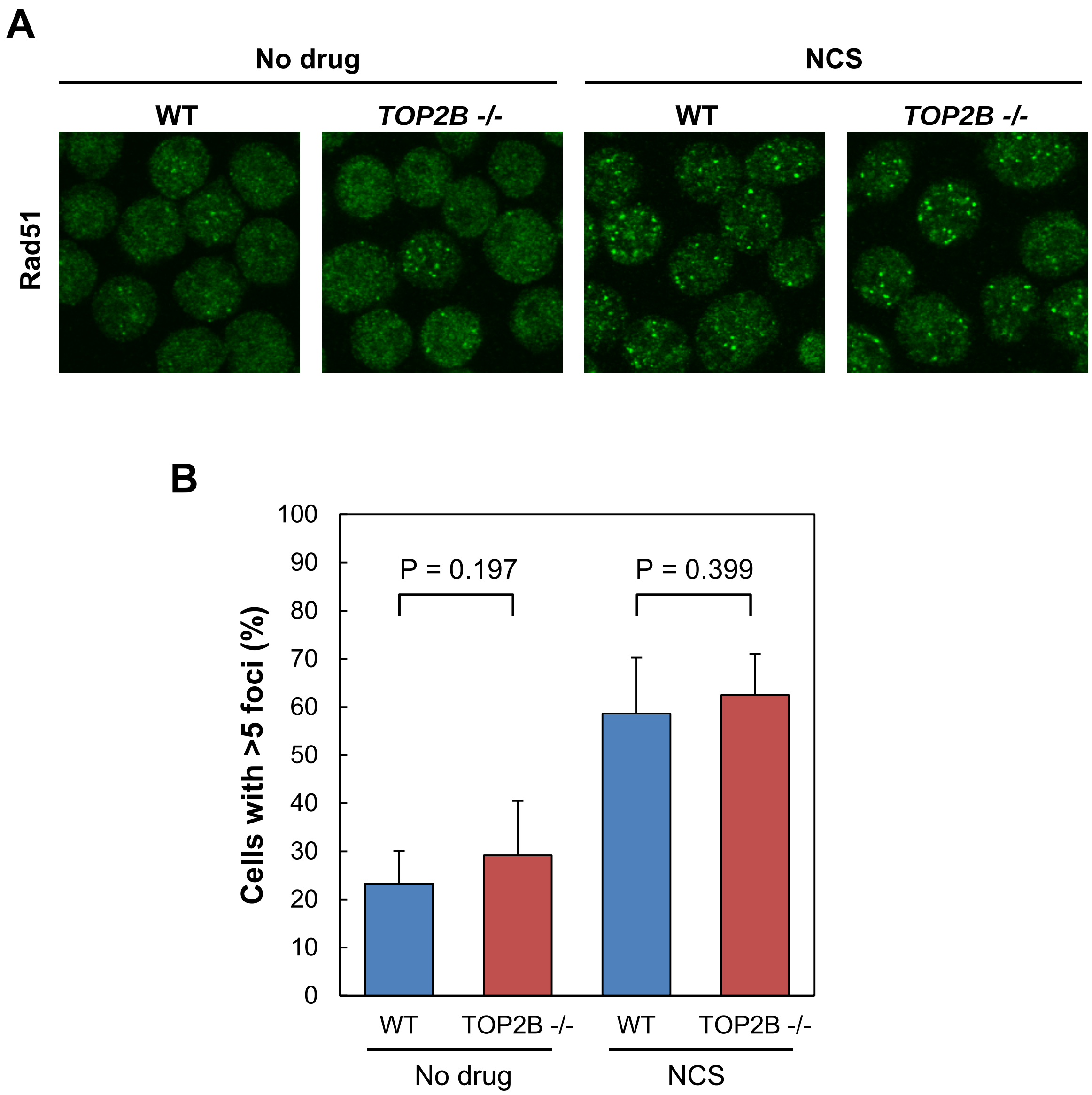

Supplementary Figure S6 Focus formation of Rad51 in wild-type and Topo IIβ knockout cells after NCS treatment. A. Wild-type (WT) and Topo IIβ knockout (TOP2B-/-) Nalm-6 cells were treated with and without 250 ng/ml NCS for 2 h and subsequently subjected to immunofluorescent analysis of Rad51. B. Cells that had more than 5 foci of Rad51 were scored, and average values with SD were calculated from 10 independent experiments.
